# The neutrophil–lymphocyte ratio and locoregional melanoma: a multicentre cohort study

**DOI:** 10.1007/s00262-019-02478-7

**Published:** 2020-01-23

**Authors:** Alyss V. Robinson, Claire Keeble, Michelle C. I. Lo, Owen Thornton, Howard Peach, Marc D. S. Moncrieff, Donald J. Dewar, Ryckie G. Wade

**Affiliations:** 1 Leeds Institute for Medical Research, University of Leeds, Leeds, UK; 2 Leeds Institute for Data Analytics, University of Leeds, Leeds, UK; 3 Plastic and Reconstructive Surgery Department, Norfolk and Norwich University Hospital NHS Trust, Norwich, UK; 4 Trinity College Dublin, The University of Dublin, Dublin, Ireland; 5 Department of Plastic and Reconstructive Surgery, Leeds General Infirmary, Leeds, UK; 6 Norwich Medical School, University of East Anglia, Norwich, UK

**Keywords:** Neutrophil–lymphocyte ratio, Platelet–lymphocyte ratio, Lymphocyte–monocyte ratio, Cutaneous melanoma, Recurrence, Biomarker

## Abstract

**Objectives:**

The neutrophil–lymphocyte ratio (NLR) is an inflammatory biomarker which is useful in cancer prognostication. We aimed to investigate the differences in baseline NLR between patients with localised and metastatic cutaneous melanoma and how this biomarker changed over time with the recurrence of disease.

**Methods:**

This multicentre cohort study describes patients treated for Stage I–III cutaneous melanoma over 10 years. The baseline NLR was measured immediately prior to surgery and again at the time of discharge or disease recurrence. The odds ratios (OR) for sentinel node involvement are estimated using mixed-effects logistic regression. The risk of recurrence is estimated using multivariable Cox regression.

**Results:**

Overall 1489 individuals were included. The mean baseline NLR was higher in patients with palpable nodal disease compared to those with microscopic nodal or localised disease (2.8 versus 2.4 and 2.3, respectively; *p* < 0.001). A baseline NLR ≥ 2.3 was associated with 30% higher odds of microscopic metastatic melanoma in the sentinel lymph node [adjusted OR 1.3 (95% CI 1.3, 1.3)]. Following surgery, 253 patients (18.7%) developed recurrent melanoma during surveillance although there was no statistically significant association between the baseline NLR and the risk of recurrence [adjusted HR 0.9 (0.7, 1.1)].

**Conclusion:**

The NLR is associated with the volume of melanoma at presentation and may predict occult sentinel lymph metastases. Further prospective work is required to investigate how NLR may be modelled against other clinicopathological variables to predict outcomes and to understand the temporal changes in NLR following surgery for melanoma.

## Introduction

The incidence of melanoma has risen faster than any other cancer worldwide [1, 2] and the status of the sentinel lymph node (SLN) is the single most important prognostic factor [3]. Whilst there are several algorithms for predicting metastases of melanoma to the SLN [4–10] their external validity is weak [4, 11–14]. Consequently, only 1 in 5 patients undergoing SLN biopsy yield a node with microscopic deposits [15]. SLN biopsy for melanoma carries an 11% risk of complication [16] and recent trials [17, 18] suggest no additional survival benefit from completion lymphadenectomy. Therefore, whilst staging the draining nodal basin remains an important goal, there is a pressing need to improve patient selection and avoid unnecessary SLN biopsies which might be achieved using host biomarkers [19].

For surgically resected BRAF V600-positive Stage III melanoma, adjuvant dabrafenib and trametinib improves survival, although discontinuation due to adverse effects is common (25%) [20]. Further, adjuvant treatment for non-BRAF-mutated tumours improves survival but again, 15% experience drug-related adverse effects and rarely, premature-death [21]. Therefore, it may be desirable to refine the selection of patients for adjuvant therapy to those at the highest risk of recurrence.

With recent advances in adjuvant therapy [22] and a rising incidence, the number of patients living with melanoma has dramatically increased. In light of the findings of the Multicentre Selective Lymphadenectomy Trial (MSLT-II) trial [19] yet more patients will be subject to surveillance rather than up-front lymphadenectomy. Therefore, early detection of recurrent disease is desirable as systemic therapies are more efficacious in patients with a lower disease burden [23]. Hence, there is an unmet need for a cheap, simple and reliable biomarker to augment the selection of patients for SLN biopsy and adjuvant therapy, and to aid in the surveillance of patients with melanoma.

In response to malignancy and for reasons that are not yet fully elucidated, the host induces a myeloid immune response (manifesting as neutrophilia and thrombocytosis) whilst suppressing the adaptive immune (lymphoid) lineage; paradoxically, this favours tumour growth, angiogenesis, and regional and distant metastasis [24, 25]. This inflammatory response is manifested in the peripheral blood neutrophil–lymphocyte ratio (NLR) [26–29], which has become an established biomarker of systemic inflammation and various outcomes in numerous cancers [30–32]. Further, the NLR has a strong association with survival in melanoma [33–39]. Currently, there is a gap in the literature concerning the relationship between NLR and outcomes in locoregional melanoma, the most prevalent form of the disease, which formed the rationale for this study.

## Methods

### Study design and patients

This is a multicentre cohort study of patients with locoregional melanoma who underwent surgery with curative intent between 2006 and 2016 in Yorkshire and the East of England, UK. A secure electronic database was prospectively completed and retrospectively augmented with blood test data from the hospitals’ electronic systems.

### Eligibility criteria

We included patients with a biopsy-proven primary cutaneous melanoma who underwent surgery (wide excision and sentinel lymph node biopsy or lymphadenectomy). Patients were excluded if no full blood count (FBC) was recorded at baseline. Patients were also excluded for any of the following reasons which are known to affect the NLR: another concurrent malignancy, active infection, pregnancy, chronic inflammatory conditions, proliferative haematopoietic disorders, pharmacological immunosuppression, multiple or occult primary melanoma, recurrent melanoma, unidentifiable or unclassifiable tumours.

### Variables

All histopathological features of the primary tumour were recorded and updated following wider re-excision. We used the FBC obtained after excision biopsy but before surgery with curative intent (i.e. WLE and SLN biopsy or lymphadenectomy) to compute the baseline NLR (absolute neutrophil ÷ absolute lymphocyte count). The platelet–lymphocyte and lymphocyte–monocyte ratios (PLR and LMR, respectively) were calculated likewise. If there were multiple blood tests acquired in this period, we used the result closest to the definitive surgery. In a nested cohort, the last available FBC (up to 28 days prior to the diagnosis of recurrence or discharge) was used to calculate changes in blood counts and their ratios over time.

### Outcomes

The primary outcome was the association between NLR and occult metastatic melanoma in the SLN, identified by histopathological assessment. Secondary outcomes included: (1) differences in the baseline NLR between localised, microscopic metastatic and macroscopic disease presentations; and (2) the changes in NLR for those who developed recurrent melanoma detected clinically, radiologically and/or by cellular techniques (histopathological assessment of a biopsy with immunohistochemistry or cytological assessment of fine needle aspirates). Patients disease-free at discharge or lost to follow-up were censored. The time to recurrence was calculated from the date of definitive surgery (SLN biopsy or lymphadenectomy) to the date of the multidisciplinary team diagnosis of recurrent melanoma.

### Statistics

There was no literature on which to base a power calculation, so this was hypothesis generating research. Data were analysed using Stata v15. Blood counts (and ratios) were skewed but lognormal, so are summarised by the geometric mean and compared using t-based methods. The threshold for NLR (2.3) was informed by previous work [34, 37–39] and selected based on the optimum Harrell C-statistic and assessment of restricted cubic splines. The association between NLR and the odds ratio (OR) for SLN metastasis was estimated using multilevel (mixed-effects) logistic regression, with random-effects carrying by the hospital, i.e. cluster. The risk of recurrence (hazard ratios, HR) was estimated using multivariable Cox regression. All covariables were selected a priori as per our protocol [6, 40–49]. Models were internally validated by lossless non-parametric bootstrapping by resampling with replacement, with 1000 iterations [50]. Confidence intervals (CI) are generated to the 95% level. The family-wise error rate was revised down according to Šidák to *p* < 0.001.

## Results

**Fig. 1:**
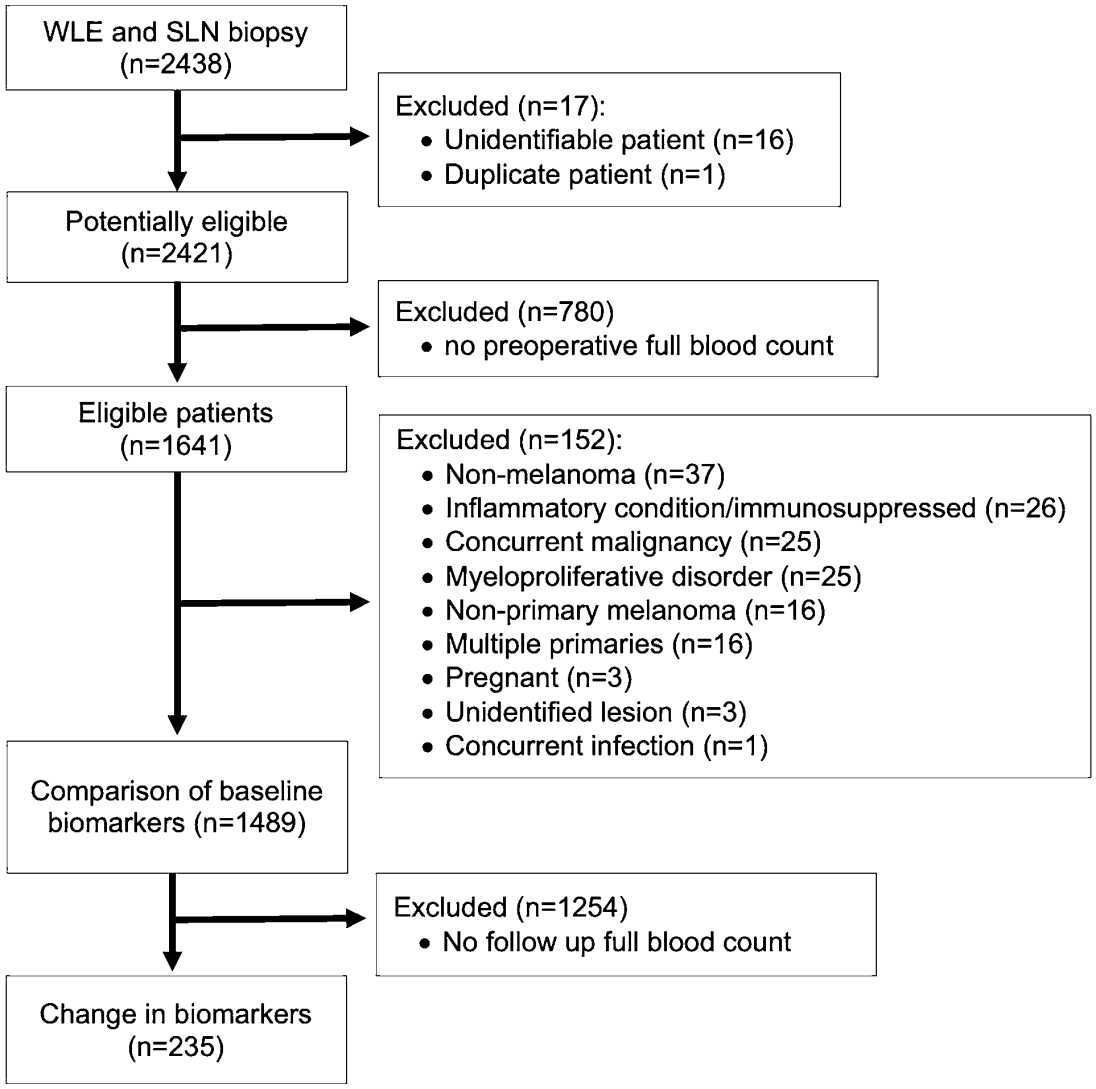
Participant flow diagram

After per-protocol exclusions, data were available for 1489 of 2438 eligible patients at baseline and a nested cohort of 235 individuals had repeated blood data for testing (Fig. 1).

### Disease status at presentation

Table 1 shows the baseline characteristics for those with localised melanoma (SLN biopsy negative), microscopic metastatic melanoma (SLN biopsy positive) and those who underwent therapeutic lymphadenectomy for palpable/macroscopic metastatic disease at presentation. There were no unexpected baseline differences between groups, except that our yield of positive SLN biopsies was higher than expected at 25%.

**Table 1:** Baseline characteristics comparing those disease-free (or censored) at final follow-up and those who developed recurrent melanoma

	Localised disease (SLN negative, *n* = 1077)	Microscopic metastases (SLN positive, *n* = 274)	Palpable disease (lymphadenectomy, *n* = 138)	*p* value
Mean age in years (SD)	63 (13)	60 (14)	65 (17)	0.004
Sex (%)				
Male	542 (50)	136 (50)	70 (51)	0.9
Female	535 (50)	138 (50)	68 (49)	
Median Breslow thickness (mm, IQR)	1.8 (1.2, 3)	2.2 (1.5, 3.5)	2.5 (1.4, 4.5)	0.001
Median Mitoses mm^−2^ (IQR)	3 (1, 7)	4 (2, 9)	7 (2, 14)	0.001
Median maximum diameter in mm (IQR)	10 (7, 14)	12 (8, 16)	12 (9, 20)	0.007
Ulceration (%)	251 (25)	72 (28)	52 (42)	< 0.001
Angiolymphatic invasion (%)	14 (3)	19 (12)	16 (20)	< 0.001
Perineural invasion (%)	17 (4)	7 (5)	2 (4)	0.9
Regression (%)	71 (17)	22 (14)	19 (23)	0.2
Microsatellites (%)	15 (4)	14 (9)	14 (25)	< 0.001
Tumour-infiltrating lymphocytes (%)				
Absent	59 (14)	33 (22)	20 (24)	0.001
Non-brisk	268 (65)	106 (70)	51 (61)	
Brisk	86 (21)	12 (8)	12 (15)	
Vertical growth phase (%)	382 (96)	142 (99)	70 (100)	0.04
Pathological subtype (%)				
Nodular	98 (9)	26 (10)	39 (28)	n/a^a^
Superficial spreading	305 (28)	104 (38)	47 (37)	
Acral	14 (12)	8 (2)	10 (7)	
Other	660 (61)	136 (50)	42 (25)	
Residual melanoma in wider re-excision (%)	51 (12)	23 (15)	25 (38)	< 0.001
Extracapsular spread (%)	n/a	16 (12)	66 (48)	< 0.001
Number of involved lymph nodes (%)				
1	n/a	81	93	n/a^a^
2		21	66	
3		3	28	
≥ 4		1	92	

^a^As we have arbitrarily grouped this data a test of proportion would not be informative

### Peripheral blood biomarkers at presentation

**Fig. 2:**
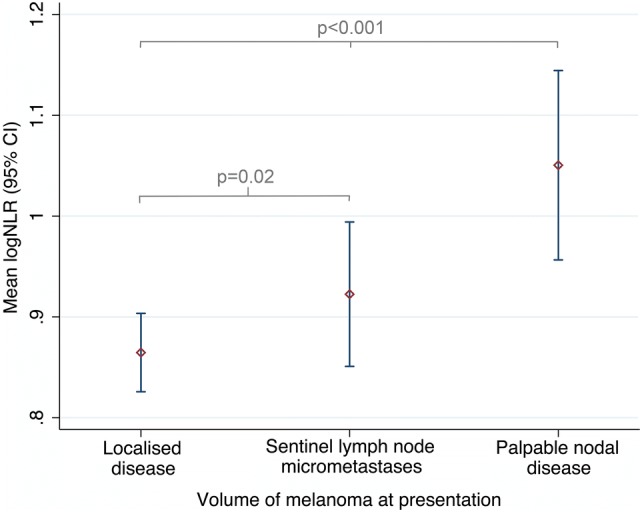
The mean logNLR (95% CI) for each group, based on disease volume at presentation. Groups were compared by ANOVA with Bonferroni correction. The logNLR is the natural logarithm of the NLR

The median time from blood test to surgery (either SLN biopsy or therapeutic lymphadenectomy) was 19 days (IQR 3, 28). Table 2 and Fig. 2 show that at presentation, the mean NLR was significantly higher in patients with palpable nodal disease [mean difference 0.2 (95% CI 0.1, 0.3), *p* < 0.001; Fig. 2] compared to others. The baseline NLR was also significantly higher in patients with microscopic metastatic melanoma in the SLN compared to those with a negative SLN biopsy, i.e. localised disease only [mean difference 0.1 (95% CI 0.1, 0.2), *p* = 0.02; Fig. 2].

**Table 2:** Blood values at disease presentation and final follow-up

	Geometric means (95% CI)							
	Leucocytes	Neutrophils	Lymphocytes	Monocytes	Platelets	Neutrophil–lymphocyte ratio (NLR)	Platelet–lymphocyte ratio (PLR)	Lymphocyte–monocyte ratio (LMR)
Disease status at presentation								
Localised (SLN negative)	7.2 (7.0, 7.3)	4.4 (4.3, 4.6	1.9 (1.8, 1.9)	0.4 (0.4, 0.4)	252 (246, 258)	2.3 (1.8, 3.1)	132 (130, 140)	4.6 (4.5, 4.8)
Microscopic metastases (SLN positive)	7.3 (6.9, 7.7)	4.6 (4.3, 4.8)	1.8 (1.7, 1.9)	0.4 (0.4, 0.5)	249 (239, 259)	2.4 (1.8, 3.3)	137 (129, 146)	4.3 (4.0, 4.6)
Palpable disease (lymphadenectomy)	7.4 (7.1, 7.8)	4.7 (4.5, 5.1)	1.7, (1.6, 1.8)	0.4 (0.4, 0.4)	255 (244, 266)	2.8 (2.0, 3.7)	153 (142, 165)	4.0 (3.7, 4.4)
* p* value^a^	0.4	0.09	0.003	0.3	0.7	< 0.001	0.003	0.001
Disease status at final follow-up								
Disease free (discharged)	7.1 (6.7, 7.6)	4.2 (4.0, 4.5)	1.8 (1.7, 1.9)	0.4 (0.4, 0.4)	243 (234, 253)	2.4 (2.2, 2.6)	138 (130, 146)	4.5 (4.2, 4.8)
Recurrent melanoma	6.8 (6.4, 7.3)	4.1 (3.8, 4.5)	1.7 (1.6, 1.9)	0.4 (0.4, 0.4)	241 (227, 256)	2.4 (2.1, 2.6)	139 (127, 151)	4.3 (3.9, 4.7)
* p* value^a^	0.4	0.5	0.8	0.5	0.8	0.9	0.9	0.4

*SLN* sentinel lymph node

^a^Derived from linear regression of lognormal data

Table 3 shows a strong univariable association between NLR and the risk of microscopic metastatic melanoma in the SLN. As a crude continuous predictor, the odds of metastatic disease in the SLN appeared to increase by 12% per unit rise in NLR [OR 1.2 (95% CI 0.1, 1.2), *p* = 0.03], compared to those with a localised disease. At a threshold of NLR ≥ 2.3 there was a statistically significant association between a raised NLR and microscopic metastatic melanoma in the SLN, whereby a NLR ≥ 2.3 increased the odds of metastasis by 30%. Resampling did not change these estimates [bootstrapped adjusted OR 1.3 (95% 1.2, 1.4), *p* < 0.001].

**Table 3:** Odds of occult sentinel lymph node metastasi**s**

	Univariable OR (95% CI)	*p* value	Adjusted^a^ OR (95% CI)	*p* value
NLR ≥ 2.3	1.2 (0.9, 1.6)	0.3	1.3 (1.3, 1.3)	< 0.001
Angiolymphatic invasion	4.1 (2.0, 8.4)	< 0.001	3.5 (1.9, 6.2)	< 0.001
Microsatellites	2.8 (1.3, 5.9)	0.008	1.7 (1.4, 2.0)	< 0.001
Mitoses per mm^2^	1.0 (1.0, 1.1)	0.001	1.1 (1.1, 1.1)	< 0.001
Breslow thickness (mm)	1.1 (1.1, 1.2)	< 0.001	1.1 (1.1, 1.2)	< 0.001
Age in years	1.0 (1.0, 1.0)	0.010	1.0 (1.0, 1.0)	< 0.001
Male	1.0 (0.8, 1.3)	0.9	1.1 (0.8, 1.5)	0.7
Ulceration	1.2 (0.9, 1.6)	0.4	1.1 (0.8, 1.5)	0.8
Regression	0.8 (0.5, 1.4)	0.5	0.9 (0.7, 1.2)	0.5
Anatomical location				
Head and neck	1 (referent)	0.002	1 (referent)	0.01
Upper limb	1.5 (0.9, 2.7)		1.5 (0.6, 3.5)	
Lower limb	2.3 (2.3, 4.0)		1.8 (0.9, 3.6)	
Trunk and genitals	2.4 (0.9, 2.7)		2.5 (1.2, 5.0)	
TILs				
Absent	1 (referent)	< 0.001	1 (referent)	< 0.001
Non-brisk	0.7 (0.4, 1.1)		0.6 (0.6, 0.6)	
Brisk	0.2 (0.1, 0.5)		0.2 (0.2, 0.3)	

*CI* confidence interval, *OR* odds ratio, *TILs* tumour-infiltrating lymphocytes

^a^Mixed-effects logistic regression with random-effects varying by the hospital cluster

### Change in NLR over time

The median follow-up was 3.6 years (IQR 2, 6 years; range 3 months to 10 years). During follow-up 253 patients (19%) developed recurrent melanoma which included 16 (13%) local recurrences, 44 (35%) nodal recurrence and 66 (52%) distant metastases; in the remaining cases the precise location of the recurrence was unclear.

Repeat (paired) blood data were available for a nested cohort of 235 individuals, all of whom had undergone SLN biopsy (80 positive, 155 negative). In this nested cohort, 86 (37%) developed recurrence after a median of 50 months (IQR 26, 75). The remaining 149 individuals were disease-free at discharge after a median surveillance of 75 months (IQR 52, 113). Table 2 shows no statistically significant difference in peripheral blood cell counts or their ratios, between those who developed recurrence and those who were disease-free at discharge. Further, in those who developed recurrence, there was no statistically significant change in peripheral blood cell counts or their ratios from baseline to the onset of recurrence.

### Baseline NLR and the risk of recurrence

**Fig. 3:**
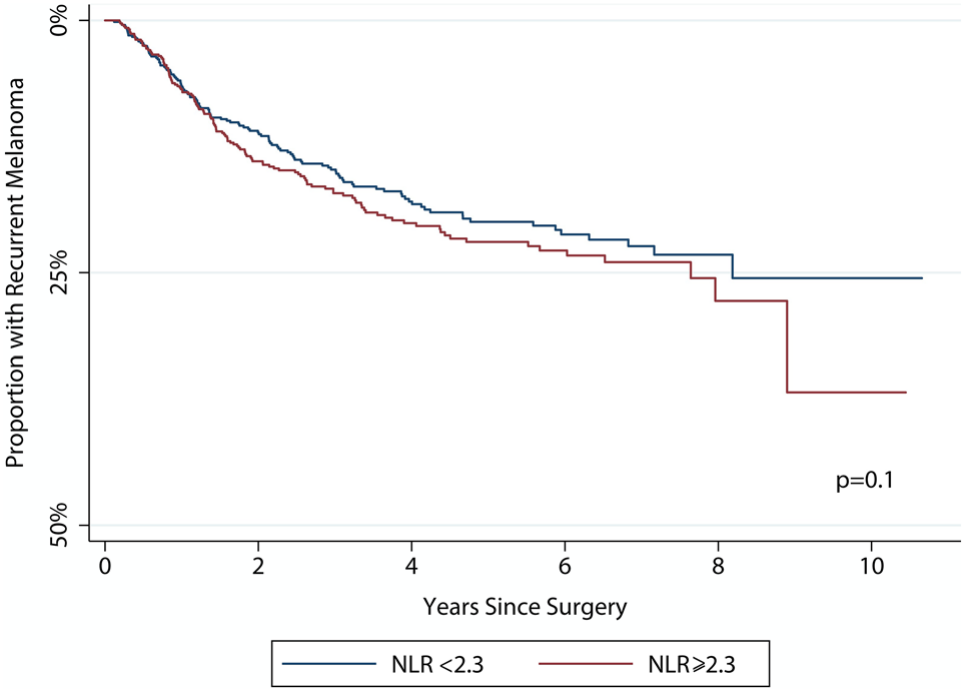
A Kaplan–Meier plot showing the development of recurrence according to the baseline NLR. The *p* value is derived from the log-rank test

Table 4 shows that the NLR was not significantly associated with the risk of recurrence in either univariable [HR 1.1 (95% CI 1.0, 1.2)] or multivariable [adjusted HR 0.9 (95% CI 0.7, 1.1)] models, which is summarised by the Kaplan–Meier plot in Fig. 3. The only variable which predicted disease recurrence was SLN positivity which increased the risk of relapse fivefold. Re-sampling did not change these estimates.

**Table 4:** The risk of recurrent melanoma

	Unadjusted risk		Adjusted^a^ risk	
	HR (95% CI)	*p* value	HR (95% CI)	*p* value
Host factors				
Neutrophil–lymphocyte ratio	1.1 (1.0, 1.2)	0.2	0.9 (0.7, 1.1)	0.2
Age	1.0 (1.0, 1.0)	0.008	1.0 (1.0, 1.0)	0.01
Primary tumour factors				
Breslow thickness	1.1 (1.1, 1.2)	< 0.001	1.1 (1.0, 1.3)	0.009
Ulceration	1.5 (1.1, 2.0)	0.003	1.1 (0.6, 2.0)	0.9
Mitoses per mm^2^	1.0 (1.0, 1.1)	< 0.001	1.0 (1.0, 1.0)	0.1
Angiolymphatic invasion	3.4 (2.1, 5.5)	< 0.001	1.4 (0.7, 2.9)	0.4
Microsatellites	2.5 (1.4, 4.2)	0.001	1.8 (0.4, 4.0)	0.2
Absence of tumour-infiltrating lymphocytes	4.9 (2.3, 11)	< 0.001	5.6 (1.6, 19)	0.008
Location				
Trunk and genitals	1 (referent)	< 0.001	1 (referent)	0.001
Upper limb	0.7 (0.5, 1.0)		0.5 (0.3, 0.9)	
Lower limb	1.4 (1.1, 1.9)		1.0 (0.6, 1.8)	
Head and neck	1.4 (1.0, 2.1)		2.4 (1.3, 4.5)	
Regional node factors				
Sentinel lymph node metastasis	5.8 (4.5, 7.3)	< 0.001	5.7 (3.8, 8.5)	< 0.001
Extracapsular spread	1.0 (0.5, 2.0)	0.9	–	–

*HR* hazard ratio, *CI* confidence interval

^a^Multivariable Cox regression with age, Breslow thickness, mitotic rate and NLR modelled as continuous variables, whilst other co-variables were handled as categorical variables

## Discussion

This study suggests that the neutrophil–lymphocyte ratio (NLR) is proportional to the volume of cutaneous melanoma at presentation. This finding supports the wider literature on other cancers which infers that the NLR represents the host response to malignancy and thus, is a reliable and personalised biomarker.

There are three published works concerning the NLR and lymph node metastases of melanoma [34, 37, 38], all of which agree with our findings that a raised NLR is associated with occult metastatic disease. Gandini et al. [34] compared absolute blood counts between SLN biopsy positive and negative individuals using rank-based methods and found no evidence of a difference; but when modelled against disease progression using Cox regression (from Stage I/II to III, i.e. when melanoma metastasises), a raised NLR was strongly predictive. This agrees with our data whereby the crude blood counts were not different between groups and highlights the power of ratios which magnify smaller differences to appreciable levels.

Lino-Silva and colleagues [38] showed that a NLR > 2 was associated with nodal metastasis; however, their study concerned acral lentiginous melanoma in the Mexican population which is a biologically distinct tumour and population, respectively. This reduces the generalisability to the majority of affected individuals who are Caucasian with nodular or superficial spreading melanoma [51]. The proportional analysis by Davis et al. [37] showed that the baseline NLR was higher in patients with a more advanced nodal substage and thicker tumours. However, they found no significant difference in the NLR between patients with macroscopic and microscopic metastatic melanoma [37]. Overall, our findings agree with the literature and suggest that a raised baseline NLR is associated with occult metastatic melanoma. Previous works investigated NLR incidentally or as a secondary outcome of interest [34, 37, 38] whereas this study adds an important dimension to the literature because the biomarker was of primary interest, analysed in a comprehensive fashion and adjusted for potential confounding variables. None-the-less, we feel that further prospective research is needed to mitigate biases of selection and information before the NLR is utilised in the management of patients with locoregional melanoma.

There is a wealth of data on haematological biomarkers in metastatic melanoma, which show that a raised baseline NLR is associated with almost twice the risk of recurrence following systemic therapy [HR 1.86 (95% CI 1.2, 2.8)] [52]; however, the literature is comparatively sparse in locoregional melanoma [34, 37–39]. Of these studies, Lino-Silva [38] is the only one to report the association between baseline NLR and the risk of recurrence. They stated that in 376 patients a baseline NLR ≥ 2 was associated with a higher risk of recurrence (28% versus 22%), although the limitations of their sample have already been discussed. Also, this apparent proportional difference was not subject to a hypothesis test (and so no effect size was offered), nor was it adjusted for important baseline confounders. These factors might explain why it differs to our finding. We add data to this important deficit in the literature concerning biomarkers in locoregional melanoma and suggest that future researchers seek to evaluate the utility of the baseline NLR in a prospective cohort of individuals with locoregional melanoma, with regular repeated measurements (including immediately after surgery) to better understand the temporal change of this valuable biomarker in melanoma.

The translational value of the NLR in the care of patients with melanoma is potentially important because the association between NLR and survival from metastatic melanoma is unequivocal [52]. The systematic review and meta-regression by Ding et al. [53] (using data from 12 studies and 3207 individuals with melanoma) showed that the NLR was strongly predictive of overall survival [HR 2.2 (95% CI 1.6, 3.0)] and disease-free survival [HR 2.2 (95% CI 1.8, 2.7)]. Recent data from our group complements this review and showed that the baseline NLR was a potentially powerful adjunct to SLN biopsy for identifying those individuals at the highest risk of death [39] who might benefit most from adjuvant therapy. Therefore, as SLN status is the best predictor of survival in melanoma, and survival is strongly associated with the NLR, our findings and those of prior studies [34, 37, 38] suggest that NLR might help to better inform treatment choices for patients in the future [19].

### Limitations

The retrospective nature of the study meant many of the patients did not have a blood test during surveillance, hence our nested cohort was only 235 patients and may have been underpowered to detect a significant difference in NLR. The median follow-up for recurrence was only 3.6 years, which would capture over 80% of disease recurrences [54] but may not identify up to 11% of late melanoma recurrences [55]. We quantified change in biomarkers by comparing the pre-operative values to the final value; however, it is possible that the NLR may normalise following surgery and therefore affect our findings, i.e. if the NLR was abnormal preoperatively but regressed to normal following surgery, before again rising with recurrence. Our study was subject to missing data but the missingness was completely at random, so unlikely to bias the outcomes. Future work could measure postoperative NLR in addition to pre-operative levels, to understand the temporal changes following surgery.

## Conclusion

The baseline NLR is associated with the volume of cutaneous melanoma at presentation. Further prospective research is needed to understand how this personalised biomarker changes following surgery for melanoma and whether it may also be used for surveillance.

## Abbreviations

CI: Confidence interval
FBC: Full blood count
HR: Hazard ratio
IQR: Interquartile range
NLR: Neutrophil lymphocyte ratio
OR: Odds ratio
SLN: Sentinel lymph node
WLE: Wider local excision
